# Informing the Co-Development of Culture-Centered Dietary Messaging in the Inuvialuit Settlement Region, Northwest Territories

**DOI:** 10.3390/nu14091915

**Published:** 2022-05-03

**Authors:** Julia Gyapay, Kanelsa Noksana, Sonja Ostertag, Sonia Wesche, Brian Douglas Laird, Kelly Skinner

**Affiliations:** 1 School of Public Health Sciences, University of Waterloo, Waterloo, ON N2L 3G1, Canada; jgyapay@uwaterloo.ca (J.G.); sonja.ostertag@uwaterloo.ca (S.O.); brian.laird@uwaterloo.ca (B.D.L.); 2 Independent Researcher, Tuktoyaktuk, NT X0E 1C0, Canada; kanelsajade15@gmail.com; 3 Department of Geography, Environment and Geomatics, University of Ottawa, Ottawa, ON K1N 6N5, Canada; swesche@uottawa.ca

**Keywords:** Indigenous health communication, Indigenous knowledge, food communication, dietary messaging, country foods, store-bought foods, Inuit, community-based research

## Abstract

Northern Indigenous communities require collaborative approaches to health communication about food that are grounded in Indigenous knowledges and cultures; however, preferences and best methods for this process remain understudied. This participatory study discusses how Inuvialuit (Inuit from the Western Arctic) knowledge and the perspectives of territorial, regional, and local dietary message stakeholders can inform the co-development of culture-centered dietary messaging to support healthy, safe, and culturally appropriate diets in Tuktoyaktuk, NWT. A community researcher in Tuktoyaktuk conducted storytelling interviews with country food knowledge holders (*n* = 7) and community members (*n* = 3), and a talking circle with local public health dietary message disseminators (*n* = 2) in June–July 2021. The lead author conducted key informant telephone and videoconference interviews with territorial and regional dietary message disseminators (*n* = 5) in June 2021. Interviews were coded and analyzed thematically. Our findings indicate that participants at all levels support increased inclusion of cultural and community perspectives about food to develop regionally and locally tailored dietary messaging. While most dietary message stakeholders wish to be involved in co-development processes, some country food knowledge holders in Tuktoyaktuk expressed a desire to lead local communications about country foods. Informed by participants’ experiences and needs, we provide recommendations for future community-led approaches to further (co-)develop and communicate effective, culturally meaningful dietary messaging that promotes Inuvialuit food sovereignty.

## 1. Introduction

Nutrition communication is a mechanism for improving a population’s nutritional well-being via the transmission of nutritional information to influence knowledge, attitudes, or behaviors [1,2]. In Canada, nutrition communication is primarily developed and disseminated federally by Health Canada via dietary guidance, information, and advice about making healthy food choices [3]. Federally-developed messaging is further transmitted and acted upon by provincial/territorial/regional governments, health professionals, academics, and non-governmental organizations to support healthy living among Canadians [3,4]. The most prominent example of Canadian dietary guidance is Canada’s Food Guide (CFG), a policy and educational tool designed to promote healthy food choices and reduce the risk of nutrition-related chronic diseases [3]. The CFG was adapted in 2007 to reflect the food systems of Indigenous peoples in Canada (“Eating Well with Canada’s Food Guide-First Nations, Inuit and Métis”), yet this Indigenous Food Guide (IFG) was criticized for adopting a pan-Indigenous approach and prescriptively focusing on food groups and portion sizes [5,6]. Overall, Indigenous-focused federal dietary guidance is greatly lacking, perpetuating dominant, Western biomedical narratives of food in Canada [7].

In the context of Indigenous communities, nutrition communication studies emphasize the importance of partnering with Indigenous peoples in the development and communication of messages [8,9]. Likewise, they recognize the importance of grounding messages in cultural and community knowledge, skills, values, and worldviews in a way that provides culturally sensitive information and approaches to foster healthy food choices [2,8,9]. This culture-centered approach helps empower communities to make healthy and safe food choices informed by both science and Indigenous knowledge, improving the effectiveness of communication efforts to initiate behavior change [10].

Health risk communication scientists working in the Canadian Arctic have called for improved participatory approaches and better inclusion of local Indigenous culture and knowledges in message development and communication about country (wild-harvested) food risks to ensure that messages are relevant, trusted, culturally appropriate, and respectful [10,11,12,13,14,15]. Despite this call, existing studies have not addressed the best methods for including local and Indigenous knowledges in such messaging. Further, it remains unknown whether involvement in the co-development of culturally relevant dietary messaging is desired by territorial, regional and local dietary message disseminators in the Canadian Arctic and if so, what these approaches and co-development processes should look like. Likewise, no studies have sought to determine desired aspects of Indigenous knowledge and local perspectives about the food system to be included in messaging in Arctic Indigenous communities, how this knowledge should be collected and utilized, and by whom.

Our transdisciplinary Country Foods for Good Health (CFGH) study addresses some of these issues in the Inuvialuit Settlement Region (ISR), a land claim area located primarily within the northern Northwest Territories (NWT). The ISR is the land of the Inuvialuit (Inuit of Canada’s western Arctic), one of four Inuit regions in Canada which collectively comprise Inuit Nunangat, the Inuit homeland [16]. Present-day governance and policymaking in the ISR are largely influenced by the terms stipulated in the 1984 Inuvialuit Final Agreement (IFA), the first comprehensive land claim agreement signed north of the 60th parallel [17,18]. The IFA does not include self-government, therefore political authority and healthcare delivery in the ISR are administered jointly by the Government of Canada and the Government of the Northwest Territories [18].

Our CFGH study includes a focus on dietary messages, which include information and advice that addresses the health benefits and risks of country food (‘country food’ refers to animals, game birds, fish, and plants harvested from the environment for human consumption; ‘country food’ is the term preferred by Inuvialuit, whereas ‘traditional food’ is often used by First Nations, and ‘wild food’ or ‘game’ may be used in policy contexts; several study participants referred to ‘traditional food’ or ‘native food’; these terms appear verbatim in the quote) and store-bought food (the terms ‘store-bought food’ and ‘market food’ were used interchangeably by study researchers and participants, referring to retail food sold in grocery stores) choices and processes (harvesting, trapping, fishing, buying, preserving, storing, preparing, cooking and consuming food) communicated by dietary message disseminators, (e.g., public health professionals, government health representatives, academic researchers, and Indigenous knowledge holders) to residents in the ISR with the goal of reducing harm and improving health. We have established that territorial and regional health departments, local health professionals, and allied health professionals in the ISR generally communicate dietary messages that are primarily informed by federal dietary guidance; this results in a lack of inclusion of Inuvialuit country food knowledges, cultural values and perspectives in current messaging [19]. In addition to public health departments, local country food knowledge holders (Elders and harvesters) also communicate dietary messaging to relatives and members of their community through the practice and sharing of traditional food skills and knowledge [19].

Our findings and current research gaps point to a need to address the best methods to collaboratively co-develop and communicate culturally relevant dietary messages among territorial, regional and local dietary message disseminators, researchers, and country food knowledge holders in northern contexts. Accordingly, the aim of this community-based participatory study was to determine how Inuvialuit knowledge and the perspectives of territorial, regional, and local dietary message disseminators, local country food knowledge holders, and the adult public (‘adult public’ refers to adult community members aged 18+ residing in Tuktoyaktuk) can inform the co-development of culture-centered dietary messaging to support healthy, safe, and culturally appropriate diets in Tuktoyaktuk, NWT and the ISR. The objectives were to (1) characterize existing gaps in culture-centered dietary messaging in the ISR; (2) identify public awareness of current dietary messages in the ISR; and (3) provide recommendations to further improve the development and dissemination of effective and culturally relevant dietary messaging for, in, and with the ISR.

## 2. Methodology and Methods

### 2.1. Research Approach

This study employed Community Based Participatory Research (CBPR) and decolonizing approaches. The CBPR approach supported active and equitable collaboration with Inuvialuit community research partners, recognizing the legitimacy of Inuvialuit perspectives and knowledge systems [20]. The decolonizing approach involved collaborative research activities with Indigenous communities focusing on Indigenous perspectives and epistemologies [21], with the aim of “uphold(ing) the pedagogical, political, moral and ethical principles that resist oppression and contribute to strategies that reposition research to reflect the unique knowledge, beliefs, and values of Indigenous communities” [22] (p. 30). Our choice of research approaches reflects our intention to support Inuvialuit self-determination, a prerequisite for Inuvialuit food sovereignty.

Following initial discussions about project directions and interests, the Tuktoyaktuk Community Corporation provided a letter of support for the CFGH project. Once funding was obtained, Gyapay involved community leaders and regional and territorial research partners from the initial stages of this study. For example, meetings with local leadership were held in person in Tuktoyaktuk in February 2020 and virtually in September 2020 to plan the study, receive feedback, and co-develop research materials. Given our inability to collaboratively complete in-person research activities during the COVID-19 pandemic, Gyapay developed a qualitative interview training toolkit and virtually hired, trained, and mentored an Inuvialuk community researcher, Kanelsa Noksana, in Tuktoyaktuk in April–May 2021. Together, Gyapay and Noksana reviewed and amended the ethics forms, interview guide questions, and research methods for clarity and cultural appropriateness. Importantly, by hiring a community researcher to lead in-person research activities, our team fostered research capacity and Indigenous self-determination for research activities in Tuktoyaktuk, which we hope to see continue post-pandemic. Research updates were shared with our research and community partners via quarterly newsletters and virtual group meetings in 2020 and 2021.

### 2.2. Participant Sample and Recruitment

Three methods were used in this study: (1) storytelling interviews (Group A) with Tuktoyaktuk country food knowledge holders (Elders and harvesters knowledgeable about the local food system and country food practices); storytelling interviews (Group B) with Tuktoyaktuk adult community members aged 18+ interested in improving messages about healthy and safe food choices in their community; (2) a talking circle with local public health dietary message developers and disseminators in Tuktoyaktuk (health professionals, community health workers); and (3) key informant interviews with territorial and regional dietary message developers and disseminators (representatives of the Government of the Northwest Territories (GNWT) Department of Health and Social Services (DHSS) and Environment and Natural Resources (ENR) in Yellowknife, representataives of the Inuvialuit Regional Corporation (IRC) in Inuvik, regional allied health professionals of the Northwest Territories Health and Social Services Authority (NTHSSA) Beaufort Delta Region in Inuvik) who had been interviewed for a previous project component in 2020. These three participant categories were selected to increase the diversity of perspectives, in light of the findings and gaps identified during the 2020 interviews.

#### 2.2.1. Storytelling Interviews

Storytelling interviews are an effective and culturally appropriate Indigenous research method that privilege Indigenous worldviews, values, and voices [23,24]. Oral storytelling is central to many Indigenous cultures, and is intricately connected with Indigenous ontologies, epistemologies, and relational ways of knowing [23,25,26]. Storytelling interviews are a useful method to resist dominant, Western research methods by honoring Indigenous voices and legitimizing Indigenous stories as a form of scientific knowledge [23,27]. We employed storytelling interviews to enact our CBPR and decolonizing approaches, bridge Western and Indigenous ways of knowing, and foster relational engagement with participants [23,27,28]. Given that our research blends Inuvialuit knowledge and public health messaging, the latter half of the storytelling interviews involved semi-structured interview questions about the desirability of including Inuvialuit knowledge in future public health messages and if so, what this process should look like.

#### 2.2.2. Talking Circles

Talking circles (also known as sharing circles) promote respectful, reciprocal, and culturally appropriate dialogue in a circular format; for many Indigenous cultures, circles are sacred and symbolize cycles in the natural world [29,30]. When sharing Indigenous knowledge, a talking circle is viewed as a more culturally appropriate research method than methods such as focus groups, given that participants have the flexibility to share stories relating to the research questions [27]. Talking circles involve introducing oneself, speaking one person at a time, listening respectfully to the person speaking, talking ‘from the heart’, and keeping what is shared in the circle in confidence [29]. We employed talking circles led by the community researcher to resist Western epistemology and research methods and to enact participatory, decolonizing research.

#### 2.2.3. Key Informant Interviews

Key informant interviews are qualitative, in-depth interviews of a non-random group of experts selected for their knowledge of their organization or the subject matter [31]. Given that participant selection is not random, a variety of key informants must be selected to obtain a nuanced understanding [31]. Key informant interviews typically employ closed- and open-ended questions and are often used in conjunction with other data collection methods to learn about an organization, program, problem, or topic [31]. We conducted key informant interviews with territorial and regional dietary message stakeholders to gain a detailed understanding of their current involvement in co-developing culture-centered dietary messaging in, with, and for the ISR and preferences for such processes in future.

### 2.3. Data Sources and Procedures

A list of potential participants for the storytelling interviews and talking circles was developed in collaboration with the primary community researcher, and supplemented via purposive, snowball sampling with another community researcher and a local health professional in Tuktoyaktuk. Gyapay developed a list of potential participants for the key informant interviews based on their prior involvement in the CFGH study, and an additional participant was invited to increase sample diversity. Participants were recruited by telephone and email.

Noksana conducted 10 storytelling interviews (A and B) in June 2021 and a talking circle with two participants in July 2021 in Tuktoyaktuk. Gyapay conducted 5 key informant telephone and videoconference interviews in June 2021. Storytelling and talking circle pilot interviews (*n* = 2) were initially completed by Noksana with Gyapay as part of the community researcher training. Open-ended questions were asked throughout the interviews, and probes were utilized to elicit further information and clarify participant responses. Storytelling interviews (A and B) lasted approximately 30 minutes each and the talking circle and key informant interviews lasted approximately 60 minutes each. Participants provided either verbal or written consent, and all interviews were audio-recorded with permission. All interviews were guided by an interview guide, developed by Gyapay and reviewed by Noksana (Supplementary Materials).

Demographic information (gender and self-identified ethnicity) was collected with participant consent to understand the relationship between demographics and preferences for dietary message development and dissemination. Inuvialuktun interpretation services were offered, but not requested by any participants. Storytelling interview and talking circle participants received a $50 grocery gift card in appreciation of their time and commitment.

The audio recordings were transcribed, reviewed, and analyzed utilizing Braun and Clarke’s guide to thematic analysis [32] and Saldaña’s first and second cycle coding methods [33], combining inductive and deductive approaches. Descriptive coding was conducted using NVivo^®^ version 12 qualitative analysis software [33,34]. Since Noksana was no longer in the community, a second community researcher returned transcribed data to country food knowledge holder participants in person and Gyapay returned transcribed data to key stakeholders by email. Member checking enabled participants to approve the publication of their quotations, further developing trusting relationships and actively engaging participants in the research analysis process [35,36,37]. Findings were reviewed with the community researcher and our territorial and regional partners and were returned to all participants via infographic posters. All audio files of the storytelling interviews were provided to the Inuvialuit Regional Corporation to honor OCAP^®^ principles, enabling the data to be owned and controlled by an Inuvialuit organization. This study received ethical approval from the University of Waterloo (ORE#42948) and a Scientific Research License (#16832) from the Aurora Research Institute.

## 3. Results

Here, we provide an overview of participant characteristics, followed by a discussion of seven major themes and sub-themes that emerged from the data (Table 1). We begin with a summary of the Inuvialuit food system and current practices of culture-centered dietary messaging in, for, and with the ISR from the perspective of all participants. We then discuss residents’ awareness of public health dietary messages in Tuktoyaktuk. We describe the successes and challenges of collaborative culture-centered dietary messaging efforts in the ISR and end with a summary of preferences and recommendations for collaboratively developing and disseminating culture-centered dietary messaging in Tuktoyaktuk and the ISR. We include participant quotations that are reflective of the overarching themes.

### 3.1. Participant Characteristics

The research involved 17 participants (Storytelling interviews A, *n* = 7; Storytelling interviews B, *n* = 3; Talking circle, *n* = 2; Key informant interviews, *n* = 5) (Table 2). Participation of men and women varied by method as did the participation of Inuvialuit and non-Inuvialuit.

### 3.2. The Inuvialuit Food System

Tuktoyaktuk country food knowledge holders described their understanding of the healthfulness and safety of country and store-bought foods, indicating their preference for country foods. Most participants recounted growing up eating country foods if they did not attend residential school, and all described healthy food as country food, as reflected by this statement:

*“Healthy foods mean traditional food, just more useful to us because we grew up with it. And I know that it works because everybody’s going back to traditional foods. Processed foods are not very good as we know, but fruits and vegetables are good too, but traditional food is the most important that we grow up with. So we’re still using it as of today.”*

(SIA6)

One participant noted that consuming country foods is both healthy and safe. They explained that the health benefits of consuming whale, particularly muktuk, and seal outweigh potential contaminant risks and that these country foods are healthier than store-bought foods:

*“…these native foods [country foods] are way more healthy to eat than store-bought food. It outweighs the contaminants that are in animals that we eat…you’re better off eating it than to not eat it.”*

(SIA2)

In contrast, most participants noted that processed store-bought foods are unhealthy and some indicated that chronic diseases, such as obesity and diabetes, stem from the consumption of unhealthy store-bought foods.

### 3.3. Dietary Challenges in the ISR

Most participants noted that climate change is challenging the safe preparation of country foods, requiring them to change the timing of harvesting and food preparation due to warmer weather. A commonly discussed example was the need to harvest beluga and prepare muktuk later in the summer to prevent spoilage and botulism, as explained here:

*“It’s really changing because summertime it gets too hot and you’ve got to do the whale a lot quicker than when it used to be kind of cold. Usually we’d keep it hanging up for two days, but when it gets too hot we leave it up for one day. And that’s no good… With the sun up 24 hours a day you’ve got to do it a lot quicker and it, and you leave a tarp on it too long it spoils right away so you’ve got to really watch because the sun is the one that really causes botulism or something on the whale and it gets really poisonous. So you’ve really got to take care of your whale.”*

(SIA7)

Two country food knowledge holders described how they became severely ill with botulism poisoning as a result of improper storage in hot temperatures, causing the muktuk to spoil. They described subsequently learning how to properly prepare muktuk safely in warmer weather and then sharing this knowledge with others, as recounted here:

*“…it did just about take my life, but I still continue to make it. You know, after a while we continued to make it and we still eat it. So it’s important, especially muktuk, like I said you know, you have to be really careful.”*

(SIA2)

Relating to store-bought food challenges, one participant noted that healthy store-bought foods are often unavailable or unaffordable in the ISR, challenging residents’ ability to make healthy food choices:

*“Because unfortunately in those smaller communities their healthiest option just isn’t an option… Like often there’s not any [store-bought foods] that aren’t unsweetened or there’s things that aren’t unsalted or you know it’s just, you just have to work with what’s there and it’s maybe not necessarily the healthiest or the best…”*

(KII4)

### 3.4. ISR Culture-Centered Dietary Messaging

Underscoring the need for the present study, several territorial and regional dietary message disseminators noted that current messaging in the NWT focuses heavily on nutrients and nutritional benefits of foods rather than adopting a more holistic and high-level perspective, as expressed in the following:

*“So messaging around food, especially from government entities, tends to be very nutrient focused. “Meat has iron, milk has vitamin D, vitamin A and calcium.” And I’m not entirely sure we need to be that specific. I think diet messaging should start, and I think it is, I think Health Canada and dietitians in Canada are starting to get on board with this, is use more general concepts when we’re talking about diet. So, what that might look like here is “country food is superior meat”, or “it’s equal or superior to store-bought meats”… You know, “eat a balanced diet that includes country food as well as plants”. Like getting a more general approach to messaging, emphasizing above all else that country food is both nutritious and safe, specifically from a contaminants perspective. That is number one thing that people always, always ask.”*

(KII2)

Regional and local dietary message disseminators expressed support for tailoring messages to specific ISR communities given their knowledge of local culture, food availability, and needs. However, territorial dietary message disseminators do not typically develop messages specific to the ISR, creating challenges with representation and inclusion of Inuvialuit culture in messaging. One participant did note that the GNWT has made efforts to better represent Inuvialuit communities, culture, and country foods in their general food security messaging after being informed of this omission by an Indigenous advisory board.

A country food knowledge holder highlighted the harm done by territorial messaging that lacked Inuvialuit input. Messages warning about contaminants in country foods have increased fears among residents about consuming muktuk and seal. Inuvialuit have advocated for messaging to be modified to reflect the health benefits of consuming country foods. This reflects a clear need for collaboration among local Community Corporations, Hunters and Trappers Committees, IRC, harvesters, researchers, and public health workers in the NWT when developing and communicating messaging to ensure that it is balanced and takes into consideration local culture and values:

*“I think it’s just important that the government have to start listening to what, you know when Elders speak about our traditional food because I’ve seen them try to put the fear in people, you know, and I felt it was really wrong at the time, you know, people wondering whether it’s still safe to eat our food, which is full of crap as far as I can see. You know, we shouldn’t be afraid to eat our native food, we shouldn’t. You know, even if they do have contaminants, it outweighs the benefits that we get from other parts of it…”*

(SIA2)

Relating to climate change, participants described how messaging typically does not address the safety of country foods themselves but rather focuses on the safety of harvesters when out hunting or fishing. Similarly, messaging from the GNWT DHSS about the safety and nutrition of country foods tends not to address climate change directly. Rather, messaging addresses harvester safety or the availability of new species in communities given changing habitat or migration patterns.

### 3.5. Current Practices of Culture-Centered Dietary Messaging

Next, drawing on all interviews, we describe the involvement of territorial, regional and local dietary message stakeholders in developing and/or disseminating culture-centered dietary messaging in, for, and with the ISR to contextualize current practices.

#### Involvement in Culture-Centered Dietary Messaging

We found that territorial and regional dietary message stakeholders from the GNWT DHSS, GNWT ENR, NTHSSA Beaufort-Delta Region, and IRC and local dietary message stakeholders from Tuktoyaktuk, including country food knowledge holders and community nutrition program coordinators, are involved in developing and/or disseminating messages about food in/for the ISR that include traditional knowledge and local perspectives (Table 3).

**Table 3:** Role of territorial (NWT), regional (ISR), and local (Tuktoyaktuk) stakeholders in the development and/or dissemination of dietary messages that incorporate community and cultural perspectives about food in/for the ISR, and approaches employed.

Dietary Message Department/Stakeholder	Role in Culture-Centered Dietary Messaging and Target Audience	Methods for Including Community and Cultural Perspectives in Current Messaging
GNWT Department of Health and Social Services (DHSS)		
Office of the Chief Public Health Officer (OCPHO), Advisor (Yellowknife)	Develops and disseminates country food consumption guidelines and health messages about contaminants in country foods to NWT public No ISR-specific messages	Consults with Indigenous community leadership to inform message development and communication based on Indigenous knowledges and preferences Example: Fish Consumption Guidance [38]
Office of the Chief Public Health Officer (OCPHO), Health professional (Yellowknife)	Develops nutrition messaging about country foods and store-bought foods to NWT public No ISR-specific messages	Develops and reviews messages with GNWT Indigenous advisory board to include Indigenous knowledge about country foods, traditional harvesting and preparation skills and determine best methods of communication Develops messages about country foods with country food knowledge holders and local health professionals across the NWT Example: Traditional Food Fact Sheets [39]
GNWT Department of Environment and Natural Resources (ENR)		
On-the-Land Unit (Yellowknife)	Develops and disseminates messaging about safe and culturally respectful country food harvesting practices to NWT public No ISR-specific messages	Works collaboratively with Indigenous governments, organizations, and other partners to include Indigenous knowledge about safe and respectful harvesting practices Example: Hunter Education Program [40]
Northwest Territories Health and Social Services Authority (NTHSSA)- Beaufort-Delta Region		
Regional allied health professionals (Inuvik)	Develops and disseminates messaging about nutrition, healthy country and store-bought food choices, and healthy cooking to ISR public through programming and client appointments ISR-specific messages	Develops and modifies messages to promote country foods and healthy store-bought food choices available in ISR communities Collaborates with country food knowledge holders to disseminate messages and organizes workshops for country food knowledge holders to share their traditional country food knowledge with the public Example: “Beaufort Delta Food Guide”, a modified Canada’s Food Guide ‘healthy plate’ poster incorporating Inuvialuit country foods (Figure 1)
Local health professionals and community health workers (Tuktoyaktuk)	Develops and disseminates messaging about nutrition, healthy country and store-bought food choices, and healthy cooking to ISR public through health promotion programming and patient assessments ISR-specific messages	Develops and modifies messages to promote country foods and healthy store-bought food choices available in ISR communities Organizes workshops for country food knowledge holders to share their traditional country food knowledge with the public Example: Promoting country food consumption during Well Child clinic visits with mothers [41]
Inuvialuit Regional Corporation (IRC)		
Health and Wellness Division (Inuvik)	Does not develop dietary messaging Supports dissemination of ISR-specific messages developed by communities	Provides opportunities for Inuvialuit to disseminate their knowledge about healthy, safe and traditional country food practices through workshops and programs in the ISR Example: Country food preparation workshops led by Elders
Tuktoyaktuk		
Tuktoyaktuk country food knowledge holders (Elders, harvesters, fishers, trappers)	Disseminates Inuvialuit knowledge about healthy and safe country food choices and safe harvesting and food preparation skills to the public ISR and Tuktoyaktuk-specific messages	Draws on personal experience and Inuvialuit knowledge learned from relatives and others in the community Example: Personal country food preparation demonstrations and harvesting trips
Tuktoyaktuk community nutrition program coordinators (health professionals and community health workers)	Develops and disseminates messaging about healthy country and store-bought food choices through recipes and cooking programming to the public ISR and Tuktoyaktuk-specific messages	Develops and modifies messages and resources to promote country foods and healthy store-bought food choices available in ISR communities Organizes workshops for country food knowledge holders to share their traditional country food knowledge with the public Example: Healthy Family Collective Kitchen program incorporating country foods in recipes [42]

At the territorial level, the GNWT DHSS develops messages in partnership with Indigenous communities and the GNWT’s Indigenous advisory board. These stakeholders provide direction as to how the GNWT should engage with communities to develop messaging, who should be involved, what messages should address, and preferred methods for message dissemination. The GNWT also consults communities to record Indigenous knowledge about harvesting and food preparation practices about specific country foods when developing country food dietary messaging. For example, the GNWT DHSS contacted harvesters and cooks across the NWT when developing the Traditional Food Fact Sheet series to include their Indigenous knowledge about country foods. Connections were facilitated by the Community Health Representatives (CHRs), trained liaisons who provide community health services in collaboration with local health practitioners.

Unlike the GNWT DHSS, the IRC does not formally gather and share community and cultural perspectives about food since they do not develop or disseminate dietary messages to beneficiaries. Rather, the IRC employs Inuvialuit and invites Elders to share knowledge about the Inuvialuit food system during programming. Thus, reflecting its larger purpose, the IRC supports Inuvialuit in directly sharing their knowledge with their community about healthy and safe food practices through cultural teachings.

At the local level, one health professional indicated that they utilize Canada’s Food Guide for First Nations, Inuit and Métis to encourage clients to incorporate country foods into their diet. Complementarily, they discussed searching online for information about which types of country food to consume for specific health profiles, so as to tailor their advice to clients:

*“So, if I’m talking with a patient that maybe needs a little bit more fibre in their diet, I can help them just Google it and we, you know, we look for credible websites and then we’ll come up with a list of foods… and so you can actually find traditional foods online that are more specific to the northern diet, right… we have access to Canada’s Food Guide, but the northern version as well. So, it includes traditional foods… there’s seal meat on there, and there’s char, and caribou, and muktuk and that kind of stuff. So, it’ll show you which part of the food guide it’s part of and then how to kind of incorporate that into your diet as well.”*

(TC2)

Importantly, local country food knowledge holders (Elders, hunters, fishers, and trappers) disseminate dietary messages in their community when sharing their knowledge about the Inuvialuit food system, most often when harvesting, trapping, fishing, preserving, storing, preparing, and cooking country foods. Participants described knowing which country foods are healthy and safe to harvest and consume by drawing on their Inuvialuit knowledge, which has been passed down to them by parents, grandparents, and other relatives, and learned from experience. Inuvialuit knowledge holders teach younger relatives how to determine the health of animals and safely harvest and prepare country foods through demonstrations and hands-on practice during harvesting trips and when preparing foods at home. As one participant explained:

*“I learn [teach] them… with my relatives, or take them out hunting or camping. And as we do down here, we invite anybody to come and watch and learn as we do.”*

(SIA6)

### 3.6. Awareness of Public Health Dietary Messages in Tuktoyaktuk

To better understand the current state of dietary messaging in the ISR, we asked about participant awareness of such messages. Some, but not all country food knowledge holders were aware of dietary messages promoting healthy and safe food in Tuktoyaktuk. Those who indicated awareness described seeing posters at the local health center or at Healthy Babies and Healthy Families programs and hearing or seeing messages about healthy food choices from doctors, commercials on TV, and Facebook posts. Importantly, one participant identified Elders as important local dietary message disseminators, highlighting the reality that dietary messages in Tuktoyaktuk are not solely developed and communicated by public health departments; Inuvialuit knowledge offers another form of dietary messaging. The participant explained:

*“And then from our knowledge too, our Elders have a lot of knowledge of that too, so don’t forget about them.”*

(SIA6)

Of the dietary messages seen and heard, some but not all included messages promoting country foods. As one participant described:

*“The doctors always say the, you know the traditional foods is more healthier to have than our processed foods.”*

(SIA6)

Similar to the country food knowledge holders interviewed, some but not all residents recalled seeing and hearing dietary messaging in Tuktoyaktuk. One referred to posters promoting healthy store-bought food choices developed by the CHR and another described hearing messages promoting eating healthy store-bought foods through the Healthy Family program and Prenatal Nutrition program, and seeing posters from Nutrition North Canada and IRC explaining how to safely prepare muktuk. Another resident described messages from the Nunavut Food Guide promoting the consumption of country foods that were shared during the Healthy Babies program. They also described messages promoting harvesting, fishing, and trapping that they had seen on the TV, radio, posters, and newspapers over the past decades from a range of sources, including the federal government, GNWT, and the Government of Nunavut. Importantly, an Inuvialuk participant noted that they receive messages about country foods primarily from their family, similar to the perspective articulated by a country food knowledge holder. One participant mentioned hearing fewer messages that encourage the consumption of country foods in Tuktoyaktuk compared to messages that encourage the consumption of healthy store-bought foods and indicated the need for further messaging that promotes country foods.

### 3.7. Collaborative Culture-Centered Dietary Messaging Successes and Challenges

#### 3.7.1. Existing Collaborations with Communities

Territorial and regional dietary message disseminators collaborate with communities about messaging in various ways. The GNWT DHSS OCPHO collaborates with community leadership when developing country food consumption guidance to determine the frequency of consumption and whether the issuing of a consumption notice is required. As one participant described:

*“So one of the important parts […is] seeking out the Indigenous traditional knowledge… for example… when we’re given data on certain contaminants and we run the quantitative risk analysis, one of the input factors in those calculations is consumption frequency. And that’s where we definitely do consult with… the relevant Indigenous communities and their representatives on that particular matter.”*

(KII1)

Several territorial and regional dietary message disseminators noted that they collaborate closely with CHRs in the ISR to seek out local perspectives and knowledge about food when developing messaging and to disseminate dietary messaging on their behalf. Nevertheless, they described numerous messaging-related challenges when working across scales. For example, one participant described encountering difficulties when country food knowledge holders disseminated dietary messaging of their choosing without input from territorial and regional counterparts, creating confusing messaging. One participant noted that high turnover rates among local health professionals, such as nurses, impede collaboration with local message disseminators. CHRs in the ISR may be better-poised as collaborators, since they have lower turnover rates compared to nurses and physicians, given that many are from the community. A regional allied health professional expressed a lack of collaboration with counterparts in other NWT regions around the development of dietary messages and resources, particularly about country foods, citing regional differences:

*“Like we, up here in the Beaufort Delta, are really unique; like we don’t do, there’s nobody else that does the same type of work that we do. So I’ve never honestly asked anybody about what they do for resource development, but I kind of don’t think there is much else being done… So sometimes we’ll reach out to each other to be like “oh does anybody have anything for this or that?”, but yeah as far as collaborating for resource development there’s not been any of that.”*

(KII4)

Several participants noted that the time, resources, and communications required to improve and develop new messaging and programs focusing on country foods and Inuvialuit knowledge are a barrier, given that local and regional dietary message disseminators are strained by other job demands. Another barrier raised was the disconnect between community, government, and academic timelines and budgets, impeding the building of trusting relationships between researchers and local, regional, and territorial dietary message disseminators to collaborate on message development. A participant expressed this challenge:

*“I think again is that collaboration from the very beginning and the conversations. I don’t think, not everyone realizes the importance of those conversations and what they mean to building relationships and doing the work. And you have to have that time to allow for that negotiation and that back and forth and the figuring things out together. And of course, on the flip side, as a challenge you know, both communities and governments have very specific deadlines or time frames for things or you know, money runs out or all of that kind of stuff. So balancing that openness, that flexibility, that building from the ground up with needing to show deliverables and progress is certainly a challenge.”*

(KII5)

#### 3.7.2. Difficulties Collecting and Communicating Cultural Food Knowledge

Participants highlighted challenges related to the reality that non-Inuvialuit professionals are often in the position of creating dietary messages for the ISR, and may lack knowledge of the local culture and food system. When seeking to include cultural knowledge in dietary messaging, some experienced resistance among Inuvialuit to communicate their knowledge in written form. Further, country food knowledge holders often have different preferences for methods of harvesting and preparing country foods, making it difficult to determine the most appropriate knowledge to communicate when developing a message. When discussing community interests in accessing country food recipes, one participant noted that it is difficult for non-Inuvialuit health professionals to develop such resources. They also highlighted the lack of cultural awareness training and mentorship available to non-Indigenous health professionals working in the ISR:

*“… I sometimes wish like as [a health professional], I wish there was just more… like training or orientation provided in regards to that because they really don’t get any introduction to that when we come into these roles… I know we have a new cultural awareness training online… but yeah or even like having someone that you can connect with when you’re in these roles to kind of guide you through it. Yeah it’s tough, it’s just kind of like when you start these positions like you’re kind of on your own to kind of like figure it out and sort it out and learn.”*

(KII4)

Regarding access to appropriate information, a local health professional noted they would benefit from access to scientific research articles that discuss the nutritional benefits of country foods compared to store-bought foods to support their work and dietary recommendations to patients:

*“… there’s a lot of studies about non-traditional foods, like the healthy ones, the unhealthy ones, but I find like there isn’t too many actual studies that I can refer to, to back up my evidence, right. A lot of the stuff is just some stuff that I’ve heard from other [health professionals] or even Elders and it does make sense. But it’s, like I wish that there were actual studies that would show us, like OK like, why is caribou that much better, like does it, you know, how much iron does it have compared to beef or, you know. What is the, you know, what are the benefits for your health from eating traditional foods versus non-traditional foods and actually looking at numbers…”*

(TC1)

### 3.8. Recommendations for Culture-Centered Messaging in the ISR

Participants made numerous recommendations for co-developing and disseminating culture-centered dietary messaging in the ISR, including who should be involved in message development and dissemination, how community and cultural perspectives should be incorporated in messaging, and which types of messages they would like to see communicated.

All participants agreed that they would like to see more Indigenous knowledge and community perspectives about country food included in future dietary messaging in the NWT and ISR. Local country food knowledge holders described the importance of promoting country foods in messaging given their nutritional and cultural benefits and the importance of transmitting country food preparation skills and knowledge to youth for cultural continuity and safety, and the teaching of Inuvialuit values. One resident explained further, saying:

*“Yes. Because it’s a part of the culture and in order for culture to continue then people need to understand the—how the food fits into it and how the culture fits into the food.”*

(SIB2)

Several participants reflected on the importance of collaborations with local dietary message disseminators and community members as a means of developing messages that are more culturally relevant and respectful. A participant summarized this sentiment:

*“Well I think it’s inherently important to work together, you know and when we have representatives from the relevant stakeholders group, then it makes for basically a product that comes out that I think is much, much better at the end of the day than you know, doing it with one set of lens as opposed to multiple lens… So this is why having that sort of more grounded and realistic understanding and you know, this understanding can only be reached in consultation with our partners, Indigenous partners”*

(KII1)

#### 3.8.1. Effective Collaborations for Culture-Centered Messaging

At the local scale, all participating residents and local health professionals agreed that the IRC and GNWT DHSS should collaborate with them or other community members when developing messages about healthy foods. One identified Elders and health professionals in the community as trusted sources. Importantly, it was recommended that IRC and GNWT DHSS communicate with communities to determine which dietary messaging projects they can support and fund.

Several country food knowledge holders felt that the GNWT should transfer leadership to communities to develop and disseminate dietary messages themselves rather than relying on prescriptive government messaging. Similarly, participants commonly recognized that collaborations with Indigenous communities are needed to shift away from the development of messages *for* the ISR to the development of messages *with,* or better yet, *by*, the ISR, with the aim of decolonizing this process:

*“I think my experience is that working collaboratively and working together to identify the key questions, challenges, concerns, all of that and then you know respond accordingly, I think has to happen with multiple knowledge systems together or needs to be grounded in Indigenous knowledge systems. I think we often kind of try to figure out how to fit it in versus starting from place as a site of meaning right, and then building outwards how we do that.”*

(KII5)

Several ideas for future collaborations were recommended by local health professionals. For example, one participant noted that increased collaborations between nurses and allied health professionals in Tuktoyaktuk are needed to improve message reception by the public. Another participant suggested holding a seminar for all CHRs in the ISR to learn more about nutritional information related to country foods, which they can then share with the public:

*“And so, I wonder if the Community Health Reps in the Beaufort Delta or the ISR were able to sit on a seminar that teaches them a little bit more about traditional foods and just gives them a few good pointers. Then they could pass that information onto the patients that they see…”*

(TC1)

Another participant proposed hiring a dietitian specializing in country foods to travel to the ISR communities to provide local health professionals with additional knowledge and resources to inform messaging. A health professional described their interest in partnering with an Inuvialuit cultural coordinator and connecting with more country food knowledge holders during the development of messaging and programming to better include Inuvialuit knowledge about country foods. Another participant explained how IRC is already doing some of this work by hiring Inuvialuit knowledge holders to deliver country food programming and messaging in a culturally appropriate way:

*“…our department seems to rely on recruiting kind of people who have a strong reputation in the communities who are around, to come join our services and we don’t really prescribe or tell them what they say, they just kind of know what to say, or they have their own bit that they’re going to say. So you don’t see us being too prescriptive with that messaging, but we know the right people to get to deliver the message.”*

(KII2)

Several participants suggested collaborating with students when developing messages to provide youth with a sense of agency:

*“And I think, like the young people need, you know, sort of they need to be included, so that it doesn’t seem like something that’s dictated to them, but something rather that they’ve participated in the development of. I mean it’s really hard to get young people to buy into something that they haven’t been part of. So, I think any time we can include not just the Elders, but you know, so the collaboration between the Elders and youth is a good strategy.”*

(SIB2)

#### 3.8.2. Collecting and Communicating Local Perspectives and Knowledge about Food

Participants highlighted the importance of acknowledging that Indigenous communities hold collective expertise about their country food system, and that they have been hubs of healthy food communication since time immemorial through the sharing of Inuvialuit knowledge about harvesting and food preparation practices. Participants stressed the importance of acknowledging the historical traumas that Indigenous peoples have faced when developing dietary messaging about food, particularly surrounding the content and quantity of information provided:

*“But I think that’s something we miss is… really creating that space for messaging is so important. And I think recognizing too… that how we share and communicate information with people who are, who’ve gone through a lot of trauma is very different… the idea that we need to be meeting people where they are, but also sharing it in a way where they get the information they need without being overwhelmed and recognizing that if you’re, you know if you’re trying to survive, you don’t want a lot of information on what the arsenic levels in moose kidneys are. You know? You need to know, ‘can I eat that kidney’?”*

(KII5)

A suggested method for territorial, regional and local dietary message disseminators to collaboratively gather Inuvialuit knowledge is to ask a question to multiple community members and then verify the content through local leadership. Incorporating questions within existing nutrition and cooking programming was also suggested as a useful method to collect community perspectives. Public engagement sessions were discouraged, given the elevated volume of ongoing community sessions about any number of topics. At the territorial level, having Indigenous guidance, (e.g., from those on the GNWT’s Indigenous advisory board) was deemed necessary for DHSS to develop effective and culturally appropriate dietary messages about country foods.

Preferred methods for gathering and sharing Inuvialuit knowledge and local perspectives in dietary messages by territorial, regional and local dietary message disseminators include: creating simple, high-level messages; incorporating visuals; delivering messages orally, in person, and through Facebook; holding meetings, events, or workshops on the land when collecting and sharing local knowledge and perspectives about food; and involving communities, especially youth, in the development and communication of messages. Participants indicated the following preferred approaches for communicating dietary messages about country foods: cookbooks, posters, and brochures with local art and photos; Facebook posts; and public service announcements on CBC radio, the CBC North TV station, and the bingo channel. Effective methods of disseminating messaging in Tuktoyaktuk included posters displayed in public locations, (e.g., schools, community halls, grocery stores, hamlet and community corporation offices, and youth centers), radio, TV announcements, and Facebook posts. A local health professional suggested creating pamphlets for the CHR to distribute at the school, community hall, and grocery stores. Several participants mentioned the importance of translating dietary messages into the local Indigenous language(s). Further, oral communication of Inuvialuit knowledge about food was noted as a culturally appropriate and effective method. Finally, a territorial government representative described the importance of taking time to develop trusting relationships, familiarizing oneself with local community protocols, utilizing data management or sharing agreements directed by community preferences, and securing funding to hire community members as mechanisms for improving the inclusion of Indigenous knowledge and local perspectives in current dietary messages.

#### 3.8.3. Communicators of Cultural Perspectives and Knowledge about Food

Country food knowledge holders identified community members, especially Elders, as the primary mechanism by which Inuvialuit knowledge about country foods should be communicated to the public, including knowledge and skills related to harvesting and food preparation. Similarly, both local health professionals and the public believed that Elders should be involved in sharing their food-related knowledge with the community. Some participants suggested that IRC, GNWT DHSS, academic researchers, and local health professionals should work with Elders, via individual interviews or a knowledge circle, to develop and review messages about country food. In contrast, a country food knowledge holder expressed dismay that the GNWT and IRC are at all involved in communicating information about country foods, explaining that it should be Elders themselves who communicate their knowledge orally and experientially, following Inuvialuit tradition:

*“To me, it’s so sad we have the government and IRC helping us to promote on what we have learnt, we could pass it on like this—like you asking me questions and I’m telling you the answers. This is the way it should be taught, face to face and to do it out there you have the means to have a smoke house and stuff. That’s the way to learn. That’s how I see it.”*

(SIA6)

Participants commonly highlighted the need for non-Indigenous government health representatives and health professionals to provide opportunities for communities to develop and deliver messaging themselves, or at minimum to collaborate with Inuvialuit stakeholders when developing and delivering messaging promoting country foods and Inuvialuit knowledge. Inuvialuit residents understand their culture, food system, and local needs, and are trusted by their community to communicate health information, as explained here:

*“I mean people trust members of their own community more than folks from outside right? …Particularly with… colonization, residential schools and all of that, you know there is a huge trust gap. So wherever possible, you know working with those champions in the communities who can be the ones delivering messages is so critical.”*

(KII5)

#### 3.8.4. Types of Culture-Centered Messages

Several country food knowledge holders expressed an interest in increased messaging about traditionally-used food preparation techniques and safe methods for country food preparation. More specifically, they identified a need for knowledge sharing about preparing whale, dried meat, and dried fish. Most advocated for such messaging to be developed and delivered by Elders through hands-on workshops with youth and interested community members, as explained here:

*“First hand, watching people or even getting knowledge from the Elders. Sitting with an Elder, you get a lot of knowledge from the Elders. Like hands on or speaking with them because you know there’s a proper way and not a proper way of doing things so you have to know how to do it from the Elders because they know what they’re doing.”*

(SIA6)

Country food knowledge holders identified youth as a particularly important target group for teaching safe country food harvesting and preparation methods, given their perceived disinterest in Inuvialuit practices and susceptibility to health risks posed by improper food preparation and preservation stemming from their relative inexperience as harvesters. Responding to this gap, another knowledge holder recommended increasing on-the-land programming and Inuvialuit food preparation workshops with students and Elders. One resident explained that youth lack exposure to sufficient country food dietary messaging in their community, and advocated for the development of new youth-targeted posters for Tuktoyaktuk, for use at the school and elsewhere.

Residents agreed that they would like to see more information about the nutritional and cultural benefits of country foods included in dietary messaging; this could include promoting country foods as healthier than store-bought foods, given their nutritional benefits and association with Inuvialuit cultural values and procurement practices:

*“I think it’s important for the message to get out there that it’s healthy, that it’s healthy fats, that’s it doesn’t contain sugars and salts and it’s not, doesn’t contain preservatives. And just those messages that these are really healthy foods, they’re from the earth, you know, and these animals give their lives to us and the hunters, the traditional hunters thank them for that… and I think that’s really important.”*

(SIB1)

Further, a health professional recognized the importance of plant knowledge in the community. They suggested creating a resource outlining the traditional uses of plants; which varieties are safe to harvest, eat and use; how to identify these varieties; and how to safely prepare them for consumption. Similarly, regional and local health professionals expressed an interest in receiving support to partner with local knowledge holders to incorporate country foods in the development of food and medicine guides as well as nutrition workshops. Participants suggested improving the inclusion of Indigenous perspectives about food in messaging, particularly relating to the cultural benefits of harvesting and consuming country foods. This would enable a more holistic approach to dietary messaging that bridges both Western scientific and Indigenous knowledge systems, as noted here:

*“I mean I think the one important one and I think some do this really well and some don’t, I think is, like identifying the importance of the food we’re talking about. You know and not just sort of a fact sheet, like not just something that says you know, “you can eat this much and blah, blah, blah”. Like you know you need more cultural context to it about why the food is important and what it means and also recognizing its holistic role in things. Again and sort of Western worldviews, you know we’re very good at separating things into you know, discrete components and so I think we missed some of that sometimes.”*

(KII5)

One concrete suggestion for engaging youth in creating locally tailored and culturally meaningful dietary messaging was to work with students at Mangilaluk School in Tuktoyaktuk:

*“I think it’s important to show the harvesters too, like we have, we’re really privileged here to have really young harvesters… And they’re out and the community knows them and they know that they go out hunting and it would be nice to see them pictured doing what they’re doing, you know. And it’s encouraging to the little guys, who look up to them and their kids as well… it’s just super positive for people to see people doing stuff here… And like I was thinking in the school, it would be a fun project for a photographer, a student photographer to go out with the harvest, the young harvesters that we have and take pictures of them harvesting and, or fishing or whatever. And you know, to do some posters with pictures and then for drawings, you know, the kids could do drawings, like those are things that attract people’s attention…”*

(SIB1)

#### 3.8.5. Messaging about Store-Bought Foods

While country foods were a key focus of discussions, participants also commonly identified the need for more ISR community perspectives and realities to be included in public health dietary messaging about store-bought foods. For example, they highlighted the need for messaging about the detrimental health effects of regularly consuming unhealthy foods, such as pop, junk food, and ready-made foods. One resident felt that sufficient messaging exists regarding the nutritional benefits of healthy store-bought foods, and health professionals identified the need for messaging promoting healthy store-bought food alternatives since produce is often unavailable or too expensive to purchase in Tuktoyaktuk. Local health professionals recommended building on successful knowledge sharing initiatives previously organized by community health workers and cooking program coordinators. For example, household visits by the CHR to share information about healthy food choices were highlighted as another useful mechanism for communication. Likewise, pop-up displays at local grocery stores with visuals of amounts of sugar and sodium found in processed foods and sugary beverages were deemed an effective way of communicating nutritional information to the public. Finally, one health professional suggested partnering with the grocery stores to create a ‘stop light’ labeling system based on healthfulness to suggest appropriate levels of consumption.

## 4. Discussion

This study was designed to inform the co-development of culture-centered dietary messaging in Tuktoyaktuk, drawing from the perspectives of territorial, regional and local dietary message disseminators, local country food knowledge holders, and interested residents. Our findings confirm the need for increased inclusion of cultural and community perspectives about healthy and safe food choices and processes in dietary messaging communicated in the ISR, particularly related to the holistic health benefits of harvesting, preparing and consuming country foods. As shown by Arctic environmental health risk communication studies and Indigenous health communication studies, our findings confirm the importance of tailoring and developing messages in partnership with communities, to ensure that they are grounded in cultural and community knowledge, skills, values, and worldviews [11,12,13,14,15,44,45].

Likewise, our findings support the need for a distinctions-based approach to messaging, acknowledging the different contexts and diversity of Indigenous peoples across geographies [45]. This contrasts with messaging approaches that are meant to serve all Indigenous peoples across a diverse region, as is presently the case for most federal and territorial dietary messaging in the NWT [45]. For example, Health Canada’s 2007 Indigenous Food Guide (IFG), “Eating Well with Canada’s Food Guide- First Nations, Inuit and Métis”, adopts a pan-Indigenous approach, overlooking the diversity of Indigenous peoples and their food systems in Canada [6]. In response, numerous population-specific IFGs and healthy food guidelines [46] have been created by Indigenous communities and health organizations in Canada, (e.g., the First Nations Health Authority’s “Healthy Food Guidelines for First Nations Communities” in British Columbia, and the Government of the Northwest Territories’ “Traditional Food Fact Sheet Series”), reflecting distinctions-based and participatory approaches to message development [39,47].

Our findings also indicate a need to increase communications and collaborations among dietary message stakeholders at all levels (territorial, regional and local), especially among Inuvialuit country food knowledge holders (Elders and harvesters), youth, the GNWT DHSS, ENR, and regional/local health professionals, to co-create regionally and locally tailored dietary messages for the ISR. This finding is consistent with other research calling for participatory message development with experts from varying backgrounds, recognizing and legitimizing Indigenous knowledge holders as dietary message disseminators [8,11,15,48]. Through this ‘two-way sharing’ of Inuvialuit and Western knowledge about healthy and safe food choices and processes, dietary message stakeholders can better learn from each other and engage in a participatory process of communication combining multiple knowledge systems [44]. An example of dietary messaging combining Inuit and Western knowledge systems is the Government of Nunavut’s 2001 “Nunavut Food Guide”, promoting country foods, healthy store-bought foods, and traditional food practices [4].

As the first study of its kind with Inuvialuit, our findings have important implications for dietary message stakeholders across the NWT. Our study advances understanding of current barriers and facilitators to participatory, culturally meaningful dietary message development and dissemination in the ISR, with the aim of informing future health communication efforts in the region. Further, this research extends our knowledge of territorial, regional and local preferences for who should be involved in the collaborative development of culture centered dietary messaging in the ISR, how such processes should take place, which types of messages are needed, and what methods may be best suited to collecting and sharing community and cultural knowledge and perspectives. Notably, our findings indicate that country food knowledge holders are the preferred communicators—through observation-based teachings—of country food harvesting and preparation knowledge and skills in Tuktoyaktuk, given their wealth of empirical and hands-on experience. This approach recognizes Inuit culture as being relationship-based and observation-based, compared to Western culture, which is information-based [49]. Therefore, we call on public health dietary message stakeholders to recognize the legitimacy of country food knowledge holders as effective dietary message disseminators and to support them in communicating dietary information in the ISR through oral, visual, and hands-on teaching, to promote Inuvialuit worldviews, culture, and values. Increasing opportunities for country food knowledge holders to share their Inuvialuit knowledge about food both honors local priorities and reflects key NCCIH recommendations for the development of culturally relevant public health messaging for northern Indigenous communities during COVID-19. The NCCIH ([45] (pp. 11–12)) recommends using ‘wise practices’—namely “Indigenous ways of knowing, principles and solutions”—to inform messaging, and adopting a strength-based approach, acknowledging that “people have the knowledge and expertise to identify and address their own concerns”. These recommendations translate to the dietary messaging context in the ISR, where continued support is required to ensure that Inuvialuit have spaces to share their knowledge about healthy and safe country food practices, for messaging to be developed for communities by communities, and for enhanced intergenerational transfer of Inuvialuit knowledge.

Despite strong participant interest in collaborative approaches to messaging, our findings highlight challenges regarding limited resources and time to develop trusting, respectful and collaborative relationships among dietary message stakeholders, particularly among government health representatives, health professionals, academic researchers, and country food knowledge holders. Informed by the culture-centered dietary messaging needs identified by participants, Table 4 presents recommendations for co-developing culture-centered dietary messaging in the ISR, organized by stakeholder group. Further, sufficient resources are required to foster trusting, respectful relationships; therefore, we call on academic researchers and federal, territorial, and regional governments to fund and support projects that foster collaborations among youth, harvesters, Elders, schools, and local health professionals to co-develop locally tailored and culture-centered dietary messages in, for, and with the ISR, as desired by communities. Given that territorial and regional governments often have limited budgets, we recommend that health professionals and government health representatives partner with academic researchers on funded research projects to support the development and evaluation of ISR culture-centered dietary messages.

Drawing on the successes of collaborative COVID-19 health communication initiatives grounded in Indigenous culture, (e.g., the co-development of COVID-19 posters by Hotıì Ts’eeda and GNWT DHSS), we recommend that NWT dietary message stakeholders partner with Indigenous health organizations such as Hotıì Ts’eeda to reduce the burden of engagement for all involved stakeholders and build on existing initiatives [45,50,51].

Given our finding that some country food knowledge holders prefer communicating dietary messages directly to their community rather than collaborating with regional or territorial public health departments, we recommend a second, more decolonized approach to dietary messaging whereby communities are supported as needed by federal, territorial and regional public health departments to take leadership in message transmission. This finding has important implications for the future of public health communication in the ISR, whereby communities shift from ‘engagement in’ to ‘leadership of’ dietary message development and dissemination, ultimately fostering Inuit food sovereignty. This aligns closely with actions outlined by Inuit Tapiriit Kanatami [52] (p. 34) in their Inuit Nunangat Food Security Strategy, calling for “Inuit-defined healthy diets that meets Inuit cultural and nutritional needs”. Health Canada’s “Brighter Futures” program is a noteworthy example of an existing federally funded program supporting Indigenous-led dietary messaging in the NWT and ISR [53]. The program funds community-led “Healthy Babies” cooking and nutrition activities for parents of young children and country food harvesting trips with Elders and youth in the ISR, promoting healthy, safe, and culturally appropriate food choices and skills [53]. Further, the school curriculum in the NWT and ISR incorporates country food harvesting, preparation, and cooking programming with youth and Elders, (e.g., GNWT ENR “Take a Kid Trapping” program and school-led country food workshops), reflecting successful partnerships for community-led food programming and dietary messaging [54].

Future research is needed with country food knowledge holders, local public health dietary message disseminators, and community members from all ISR communities to compare culture-centered dietary message perspectives and priorities across the ISR, in addition to other regions in the NWT. Furthermore, there is a need for research to examine the perspectives of federal dietary message disseminators, academic researchers, and ISR youth regarding the development of culture-centered messaging to establish a greater understanding of their experiences and needs. Finally, further research is needed to evaluate participatory, culture-centered dietary messaging initiatives in the ISR and NWT to determine messaging effectiveness as well as barriers and facilitators experienced by stakeholders, with the goal of improving communication policies and practices.

Our recommendations for the collaborative development and dissemination of ISR-tailored culture-centered messaging, especially messaging promoting country foods that bridges both Western scientific and Inuvialuit knowledge, have important implications for the ISR, NWT, and Inuit Nunangat given the identified interest in more culturally meaningful messaging grounded in local and regional culture and knowledge. This research makes several noteworthy contributions to Arctic health communication literature by providing a new understanding of preferences for how culturally inclusive dietary messaging should be (co-)developed in the ISR, by whom, and which topics should be addressed to support healthy, safe, and culturally appropriate food choices and processes.

## 5. Conclusions

This participatory study employed a combination of Indigenous and Western qualitative research methods to describe how the perspectives and Inuvialuit knowledge of territorial, regional, and local dietary message stakeholders can inform the (co-)development of culture centered dietary messaging to support healthy, safe, and culturally appropriate diets in Tuktoyaktuk, NWT. As the first study of its kind examining the best methods for culture-centered dietary messaging in the ISR, our findings confirm the need for increased inclusion of cultural and community perspectives about food for the development of culturally inclusive, regionally, and locally tailored dietary messaging. Our study provides a new understanding of territorial, regional and local preferences for the (co-)development of culture-centered dietary messaging in, for, and with the ISR and offers recommendations for future collaborations and independent, community-led dietary message initiatives, promoting Inuvialuit food sovereignty through effective, culturally meaningful health communication.

## Figures and Tables

**Figure 1:**
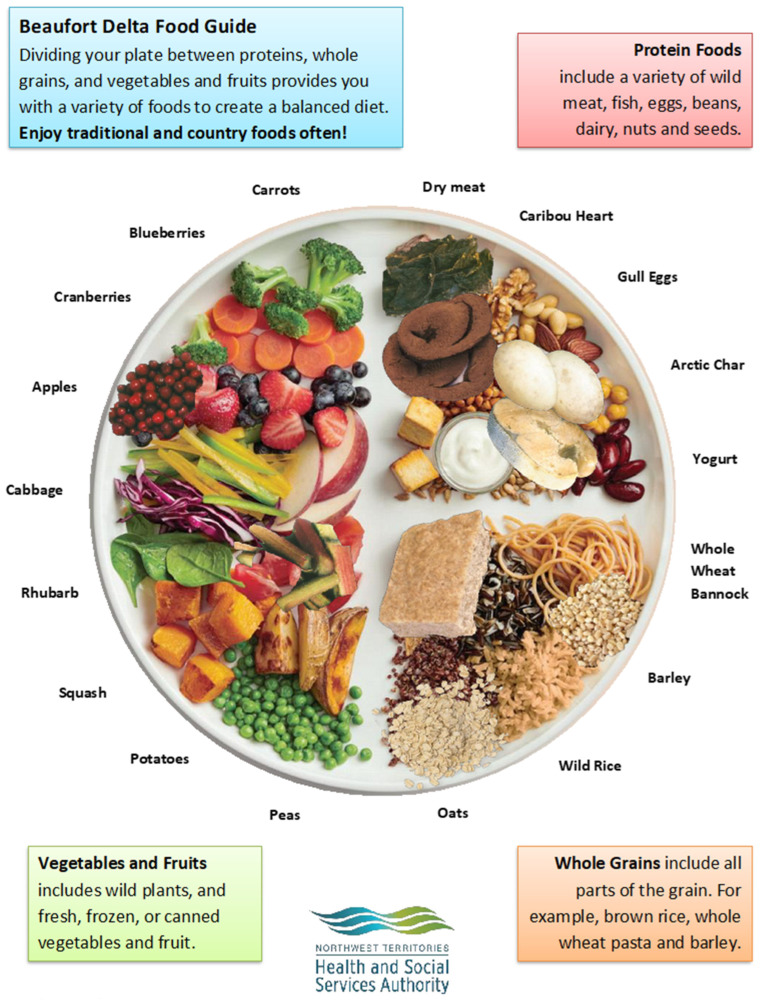
Beaufort Delta Food Guide [43].

**Table 1:** Summary of major themes and sub-themes emerging from multiple research methods.

Themes	Methods *
3.2. The Inuvialuit Food System	SIA
3.3. Dietary Challenges in the ISR	SIA, SIB, TC, KII
3.4. ISR Culture-Centered Dietary Messaging	SIA, SIB, TC, KII
3.5. Current Practices of Culture-Centered Dietary Messaging 3.5.1. Involvement in culture-centered dietary messaging	SIA, SIB, TC, KII
3.6. Awareness of Public Health Dietary Messages in Tuktoyaktuk	SIA, SIB
3.7. Collaborative Culture-Centered Dietary Messaging Successes and Challenges 3.7.1. Existing Collaborations with Communities 3.7.2. Difficulties Collecting and Communicating Cultural Food Knowledge	TCC, KII
3.8. Recommendations for Culture-Centered Messaging in the ISR 3.8.1. Effective Collaborations for Culture-Centered Messaging 3.8.2. Collecting and Communicating Local Perspectives and Knowledge about Food 3.8.3. Communicators of Cultural Perspectives and Knowledge about Food 3.8.4. Types of Culture-Centered Messages 3.8.5. Messaging about Store-Bought Foods	SIA, SIB, TC, KII

* SIA = Storytelling Interviews A; SIB = Storytelling Interviews B; TC = Talking Circle; KII = Key Informant Interviews.

**Table 2:** Participant characteristics and reference codes categorized by research method.

Method	Number of Participants (*n*)	Gender		Self-Identified Ethnicity		Stakeholder Type	Reference Code *
		Female	Male	Inuvialuit	Non-Inuvialuit		
Storytelling interviews A	7	*n* = 2	*n* = 5	*n* = 7	*n* = 0	Tuktoyaktuk country food knowledge holders (harvesters and Elders)	SIA 1–7
Storytelling interviews B	3	*n* = 3	*n* = 0	*n* = 1	*n* = 2	Tuktoyaktuk community members aged 18+	SIB 1–3
Talking circle	2	*n* = 1	*n* = 1	*n* = 1	*n* = 1	Tuktoyaktuk health professionals and allied health professionals	TC 1–2
Key informant interviews	5	*n* = 3	*n* = 2	*n* = 0	*n* = 5	Territorial (GNWT DHSS and ENR) and Regional (IRC and NTHSSA Beaufort-Delta) dietary message developers and disseminators	KII 1–5

* SIA 1–7 (Storytelling interviews A, participants 1–7); SIB 1–3 (Storytelling interviews B, participants 1–3); TC 1–2 (Talking circle, participants 1–2); KII 1–5 (Key informant interviews, participants 1–5).

**Table 4:** Recommendations for collaboratively developing dietary messages in, for, and with the Inuvialuit Settlement Region (ISR), by stakeholder group.

Dietary Message Stakeholders	Recommendations for (Co-)Developing Culture-Centered Dietary Messaging in the ISR
GNWT Department of Health and Social Services (DHSS)	
Office of the Chief Public Health Officer (OCPHO)	Collaborate with local health professionals, country food knowledge holders, and researchers to develop culture-centered and ISR-tailored messaging, incorporating Inuvialuit knowledge of country food processes and climate change adaptation considerations Fund and support dietary message development and communication projects led by communities, (e.g., student-harvester country food photo project to design posters). Partnerships with academic researchers can provide funding sources to support and facilitate such projects Develop and deliver a country food nutrition training workshop for regional and local health professionals and community health workers in the ISR
GNWT Department of Environment and Natural Resources (ENR)	
On-the-Land Unit	Collaborate with local health professionals, country food knowledge holders, and researchers to develop culture-centered and ISR-tailored messaging, incorporating Inuvialuit knowledge of country food processes and climate change adaptation considerations Fund and support dietary message development and communication projects led by communities, (e.g., country food preparation workshops led by Elders, traditional edible plant identification resources)
Northwest Territories Health and Social Services Authority (NTHSSA), Beaufort-Delta Region	
NTHSSA Beaufort-Delta Region administrators	Develop cultural training resources and mentorship opportunities with local country food knowledge holders for non-Inuvialuit (allied) health professionals Improve local health professionals’ and community health workers’ access to scientific information about the nutritional benefits of country foods through communications with researchers and the GNWT DHSS
Regional allied health professionals (Inuvik)	Increase partnerships with local country food knowledge holders and cultural coordinators to deliver dietary messaging and nutrition programming about country foods Develop a country food position to advise dietary message development in the ISR Establish communications between regional allied health professionals to share dietary message resources and develop partnerships across the NWT
Local health professionals and community health workers (Tuktoyaktuk)	Collaborate with other local health professionals and community health workers across the ISR, local leadership, schools, and Elders when developing dietary messages Establish communications between regional allied health professionals to share dietary message resources and develop partnerships
Inuvialuit Regional Corporation (IRC)	
Health and Wellness Division	Increase country food harvesting and preparation workshops and programs led by Elders, especially for youth Fund country food harvesting and preparation workshops and programs led by Elders
Community of Tuktoyaktuk	
Tuktoyaktuk country food knowledge holders (Elders, harvesters, fishers, trappers)	Increase country food harvesting and preparation workshops and programs led by Elders, especially for youth Collaborate with local health professionals and cooking programs to deliver hands-on workshops on the land

## Data Availability

Data is contained within this paper, and no further information about qualitative data can be shared due to ethical/privacy reasons as we worked with vulnerable communities.

## Supplementary Materials

https://www.mdpi.com/article/10.3390/nu14091915/s1, Interview Guide S1: Sample interview guide from Storytelling B interviews with Tuktoyaktuk community members
