# Supplementary material for: Informing the Co-Development of Culture-Centered Dietary Messaging in the Inuvialuit Settlement Region, Northwest Territories
Source: Nutrients. 2022 May 3;14(9):1915. doi: 10.3390/nu14091915 (PMC9099519; doi:10.3390/nu14091915)
Supplement: Supplementary file 1 [file nutrients-14-01915-s001.zip › nutrients-1639517-supplementary.pdf]

Supplementary Material. Sample interview guide from Storytelling B interviews with Tuktoyaktuk community members

Interview questions
1. **Have you heard messages encouraging you to eat healthy store-bought foods or country foods in Tuktoyaktuk?**<br>*Prompt: advice about healthy food choices you were given by health workers in town, during cooking programs, on Facebook or on posters you've seen in town*<br><br>If yes:<br>• What was the message about? Who was the message from? How did you hear/see it?<br>• Did the message include your Inuvialuit knowledge about country food? If yes:<br>• How did this message include your traditional knowledge?<br>• Did you like the message that included your traditional knowledge?<br>• Is there anything you didn't like about it?<br><br>2. **Do you think Inuvialuit knowledge about country food should be included in messaging promoting healthy food choices in our community?**<br><br>If yes:<br>• Why?<br>• What traditional knowledge about food would you like to see shared in future messages?<br>*Prompt: how to respectfully harvest or prepare foods, how to safely prepare foods, nutritional benefits of country foods, uses of country foods, importance of food for cultural health, mental health*<br>• How would you like to see this knowledge shared?<br>*Prompt: Inuvialuit art? Inuvialuktun translations? Photos and stories from locals*<br>• Who should communicate this knowledge?<br><br>If no:<br>• If you feel comfortable, can you share why not?<br><br>**3. What information about store-bought foods would you like to see shared in future messages?**<br>*Prompt: nutritional benefits of healthy store-bought foods, how to cook with fruits and vegetables, how to choose healthy store-bought foods on a budget*<br><br>If yes:<br>• How would you like to see this information shared?<br>*Prompt: Inuvialuit art? Inuvialuktun translations? Photos and stories from locals?*<br>• Who should communicate these messages from our community?<br>If no:<br>• If you feel comfortable, can you share why not?<br><br>**4. Do you think the Inuvialuit Regional Corporation and GNWT Department of Health and Social Services should develop messages about healthy food choices in partnership with you and others who hear and see these messages in our community?**<br><br>If yes:<br>• What should this process look like?<br>• What would help this process work well?<br>• Who in the community do you think should be involved?<br>If no:<br>• If you feel comfortable, can you share why not?<br><br>5. **Is there anything else you would like to add about how you think messaging about healthy food can be improved?**
